# The common yet enigmatic activity of histone tail clipping

**DOI:** 10.1016/j.jbc.2025.110239

**Published:** 2025-05-15

**Authors:** Elizabeth M. Duncan

**Affiliations:** Department of Biology, University of Kentucky, Lexington, Kentucky, USA

**Keywords:** chromatin, histone, clipping, proteolysis, cathepsin L, mmp-9, trypsin, mmp-2

## Abstract

Histone proteolysis is sometimes described as an extreme posttranslational modification (PTM), as it removes both a stretch of histone sequence and any PTMs that were previously added to it. Such an acute and significant loss of information could trigger many different downstream effects, making it attractive as a mechanism for rapid gene silencing or activation. However, protease activity is challenging to study and is often treated like background noise that is best kept as low as possible. As both histones and protease activity are highly abundant in most cells, evidence of proteolysis of histone tails—a.k.a. histone clipping—has often been dismissed as nonspecific noise. Yet over the past decades there have been studies suggesting this activity should not be ignored, that it may represent a rare but relevant process with important roles in cell biology. Here, I review the key studies that both support this argument and raise additional questions about the mechanisms and functions of histone clipping.

## Overview

Proteolytic cleavage of histone proteins, particularly the “clipping” of their accessible tails, is a phenomenon that has drawn intermittent attention from chromatin researchers over the last several decades. Although many scientists have likely observed evidence of this activity in their histone preparations, most will choose to dismiss it as a processing artifact or else too troublesome to pursue. The latter is certainly understandable: Studying histone proteolysis, or the proteolysis of any protein, is not straightforward. In fact, biochemists typically strive to *inhibit* protease activity in their experiments, since the process of busting cells open to extract their proteins also exposes them to enzymes that will destroy them. This makes it tricky to distinguish between *bona fide* endogenous protease activity and the spurious effects of imperfect protease inhibition during processing. In addition, proteases usually have many different substrates, making it difficult to evaluate the effects of a single cleavage event. In other words, some skepticism about the validity and *in vivo* relevance of histone proteolysis is undoubtedly warranted; who wants to spend time and money chasing an artifact?

**Figure 1:**
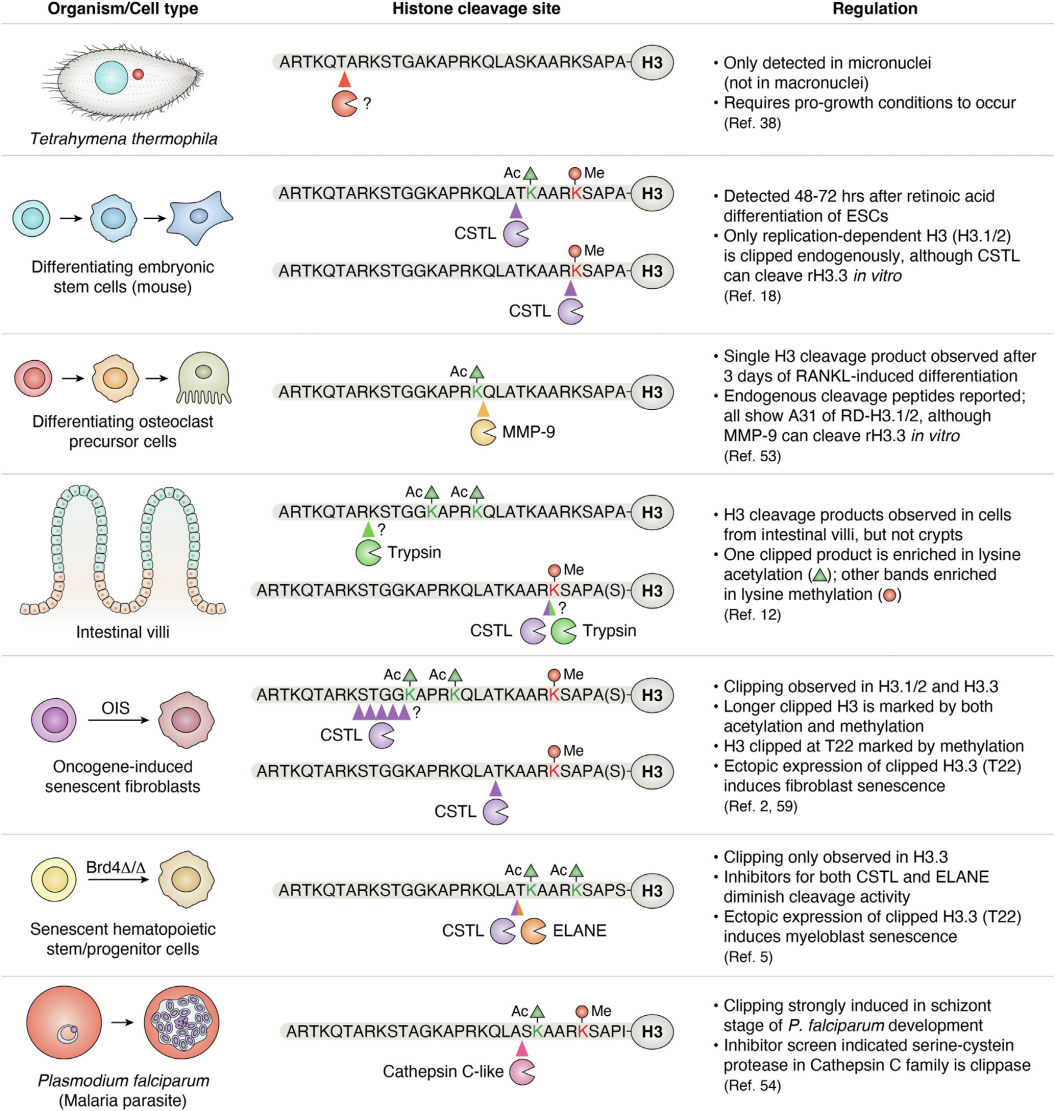
**Histone clipping occurs in divergent organisms yet exhibits common features.** A summary of some of the organisms and cell types in which histone H3 clipping has been described, their major sites of cleavage, enzymes responsible (if known), and cell/chromatin states that regulate the activity. Abbreviations: H3 (histone H3), ESCs (embryonic stem cells), RA (retinoic acid), CSTL (Cathepsin L), Ac (acetylation), Me (Methylation), rH3.3 (recombinant histone H3.3 protein), RD (replication dependent), H3.1/2 (histone H3.1 or histone H3.2 protein), MMP-9 (matrix metalloproteinase 9), RANKL (Receptor activator of nuclear factor kappa-Β ligand), A31 (alanine at position 31 in the H3 protein sequence), T22 (threonine at position 22), OIS (oncogene-induced senescence), Brd4 (bromodomain-containing 4 gene), ELANE (neutrophil elastase).

Despite these concerns, the relatively small body of literature on histone proteolysis has suddenly grown in the last few years (1, 2, 3, 4, 5, 6, 7, 8, 9, 10, 11, 12, 13). These studies have expanded the number of cell types, organisms, and contexts in which histone clipping is observed (Fig. 1), adding support to the hypothesis that clipping occurs *in vivo*, by regulated mechanisms, and triggers significant consequences. As several previous reviews have done an excellent job describing the various types of histone proteolysis and contexts in which they occur (14, 15, 16, 17), here I will focus on studies that examine whether histone proteolysis is regulated and if its action plays a regulatory role in cell biology. Having spent several years studying histone clipping during my own graduate training (18), I will also provide some additional details about my own experiments that illustrate how I addressed my initial skepticism about the “mystery bands” in my data. Finally, I consider where the histone clipping field currently stands, pose some questions raised by the findings of more recent studies, and propose some possible future directions that may help uncover new mechanistic details and *in vivo* functions of histone clipping.

## What is histone “clipping”?

Histones are proteolyzed through multiple mechanisms and in different contexts, *e.g.*, total degradation during apoptosis *versus* endoproteolytic cleavage at a defined site (19). In this review, I will focus on the latter and particularly on examples in which the cleavage site is in the amino-terminal tail of the histone protein, a.k.a. “histone clipping”. In the chromatin field, we use this more familiar term to evoke the image of a histone’s more accessible tail being rapidly and precisely separated from its globular core, likely while the histone is still embedded in the chromatin fiber. Clipped histones are fairly easy to detect by immunoblot using appropriate histone antibodies, as their mass is significantly lower than that of their full-length precursors but usually still in the range of smaller histones, *e.g.*, histone H4.

## Histone proteases as gene activators

In the 1960s and 1970s, several groups reported evidence of histone clipping in chromatin isolated from tissues such as calf thymus, rat liver, and (nucleated) chicken erythrocytes (20, 21, 22, 23, 24, 25). Rather than dismiss these observations as noise, they questioned why histones extracted from the nuclei of some tissues, *e.g.*, *Xenopus* liver and carp erythrocytes, displayed more proteolysis than those extracted from comparable cell types and animals, *e.g.*, *Xenopus* and trout erythrocytes (23, 25). Researchers observed these differences even when the nuclei were isolated in parallel using the same protocol and in the presence of protease inhibitors (21, 23, 25). Some of these scientists leaned toward the conclusion that histone proteolysis occurs *in vivo* within the nuclei before isolation; others concluded that some nuclei are more easily damaged during isolation than others, allowing proteases from the cytoplasm to access the chromatin during extraction. Although the latter view largely prevailed in the succeeding decades, some unresolved questions still lingered: why did the nucleated erythrocytes of different species exhibit significantly different amounts of histone proteolysis, with some showing none at all? Why was there still protease activity associated with some nuclei, even when they appeared undamaged and all evidence of cytoplasm washed away?

In addition to these methodological questions, some scientists were intrigued by the potential for histone proteolysis to operate as a mechanism of gene de-repression. Specifically, the degradation of histones presented a way for cells to release them from DNA, an association that was known to be a major barrier for the transcription machinery (22). Experiments performed in this era using a heterokaryon model (HeLa cells fused to chicken erythrocytes) provided some indirect support for this hypothesis. The fusion of human HeLa cells with chicken erythrocytes normally causes dormant erythrocyte nuclei to reactivate, opening their chromatin and initiating the synthesis of both DNA and RNA (26). However, researchers investigating the mechanisms of this reactivation found that dormant erythrocyte nuclei could not be reactivated if they were treated with certain protease inhibitors (27, 28). These data not only fit with a model in which histone proteolysis mediates gene de-repression (a.k.a. activation) but also filled a conceptual gap at a time when there were no other “alternative mechanisms for the dissociation of histones…from DNA *in vivo*” (22). Histone degradation, as long as it could be proven to occur in the nucleus prior to experimental manipulation, pointed to a possible mechanistic solution to this problem. Yet confirming that histone proteolysis occurred in nuclei before tissue harvesting or cell fractionation was not easy, leading many researchers in this era to conclude that the “role of the proteases in active cells remains uncertain” (25).

## Histone clipping in *Tetrahymena* micronuclei

Many of the early studies on histone proteolysis used nuclei isolated from vertebrate cells or tissues, as these were common sources of material for biochemical studies at that time. However, some scientists began pioneering the use of an invertebrate model system to investigate the role of histone proteins in regulating gene expression. Noting that the fundamental structure of chromatin is highly conserved between species, and even across domains, these researchers realized that the two distinct nuclei of the ciliate *Tetrahymena thermophila* could help them identify chromatin features that distinguish active *versus* silent regions of the genome (29). The two nuclei of these single-celled ciliates are not only physically separable by differential centrifugation but also exhibit opposite levels of gene expression: the genome of the larger macronucleus is actively transcribed whereas that of the smaller micronucleus is relatively silent (29). This simple approach of separating these two distinct nuclei and comparing their chromatin was made famous by David (Dave) Allis, who used it to characterize several of the posttranslational modifications (PTMs) that distinguish histones in actively transcribed macronuclei from those in silent micronuclei and to identify some of the enzymes that add them (30, 31, 32, 33, 34, 35, 36, 37). What is less well known is that Dave also published a foundational study on histone clipping during his time as a postdoc in the Gorovsky lab (38); in fact it was, in Dave’s recollection, his “only paper to be accepted without any revisions” (C.D.A., personal communication).

The first step in discovering histone clipping in *Tetrahymena* was to optimize the extraction of histones from their nuclei. Several early papers characterizing the histones of *Tetrahymena* nuclei had found there was very little histone H1 or H3 in micronuclei (29, 39, 40), a finding that was puzzling since other assays suggested their chromatin organization was very similar to that of the macronuclei (which contained abundant histone H3). After addressing some technical issues that were preventing efficient histone extraction from the dense micronuclei, Dave observed that they contained two versions of histone H3: one that migrated “fast” (H3^F^) and one “slow” (H3^S^) in acrylamide gels (41). This improvement was a critical step for many discoveries to come, as it allowed a less biased comparison of macro and micronuclear histones. However, it did not immediately reveal what caused the faster migration of the micronuclear-specific H^F^ protein.

The key experiment that demonstrated H3^F^ was a clipped form of H3^S^ was done in a subsequent study (38). First, Dave incubated *Tetrahymena* in simple Tris buffer spiked with radioactive ^3^H-leucine, conditions sufficient for new protein translation but not “growth,” *i.e.*, cell proliferation. He then isolated their micronuclei, extracted their histones, separated them using two-dimensional (2D) gel electrophoresis, and stained for total protein. As usual, Dave observed bands for both the H3^F^ and H3^S^ proteins. However, only the H3^S^ band was labeled with ^3^H-leucine (38), suggesting that an hour of labeling in non-growth conditions was sufficient to translate the slower-migrating H3^S^ histone but did not allow generation of the faster-migrating H3^F^. Importantly, this finding supported the hypothesis that H3^F^ is created in *Tetrahymena* cells as part of a regulated process, rather than incidentally occurring during nuclear isolation and histone extraction. Dave and his colleagues further hypothesized that H3^F^ was likely a processed form of H3^S^ and that this processing required growth conditions and/or cell division to occur. To test this idea, they set up a pulse-chase experiment: after again labeling *Tetrahymena* proteins with ^3^H-leucine in non-growth conditions (1 h in Tris buffer), they divided the culture into three groups: (1) no chase, (2) chase for 10 hours in non-growth conditions *i.e.*, Tris buffer, and (3) chase for 10 hours in growth conditions *i.e.*, in normal growth media.

As in their first experiment, Dave and his colleagues did not detect any radioactive H3^F^ protein in the histone prep from the “no chase” condition (group 1). However, almost all radiolabeled H3 protein in the third group, *i.e.*, growing *Tetrahymena* culture, migrated as H3^F^. These results strongly supported the hypothesis that H3^S^ is a precursor to H3^F^ and that growth conditions and/or cell division is required for H3^F^ formation (38). Dave and his colleagues then asked if the conversion of H3^S^ to H3^F^ is caused by proteolytic processing, a.k.a. histone clipping. Using both an *in vitro* proteolytic mapping approach and automated amino acid sequencing, they not only confirmed this was very likely but also identified the site of cleavage as between amino acids six (threonine, T) and seven (alanine, A) in the H3 N-terminal tail (38). Notably, this cleavage removes a smaller peptide than what had been reported in vertebrate cells and tissues. Nonetheless, these data show clear evidence of growth-regulated H3 clipping in *Tetrahymena*, strengthening the validity that it could occur *in vivo*.

## Histone clipping during embryonic stem cell differentiation

More than 20 years after Dave Allis published his discovery of H3 tail clipping in *Tetrahymena* micronuclei, I was doing my graduate work in his laboratory when I noticed a similar band of faster-migrating histone H3 in samples from differentiating embryonic stem cells (ESCs). When I showed Dave these unexpected findings, he was “so jazzed!” Embarrassingly, I did not appreciate the depth of his excitement at the time, as I had not yet read his paper on histone clipping in *Tetrahymena* (38). In the two decades since it was published, the chromatin field (including the Allis laboratory) had largely focused on understanding the connection between histone PTMs and gene expression. There was very little published on histone clipping in those years, despite Dave’s intriguing data suggesting that it may be regulated by the cell cycle. My unexpected finding in differentiating ESCs reignited Dave’s fascination with histone clipping, particularly as my initial data hinted that the activity might be developmentally regulated in mammalian cells.

My early graduate work had focused on the binding of Polycomb group proteins to histone H3 lysine 27 methylation (H3K27me) during ESC differentiation (42). As part of this project, I extracted histones from differentiating mouse ESCs at 24 h intervals. When I used these extracts to generate immunoblots and probe them with antibodies recognizing various modified histones (*e.g.*, anti-H3K4me3 or anti-H3K9me3), most showed no appreciable change in global modification levels. However, I noticed that some antibodies (*e.g.*, anti-H3K27me2) consistently detected a faster migrating H3 band at days two and three post-differentiation (18). Initially, I assumed these mystery bands must be technical artifacts. But after ruling out several possible mechanisms through which such artifacts could be created, I began to think the more interesting hypothesis—that these bands were the product of an *in vivo* protease activity that was induced during a specific window of stem cell differentiation—was worth exploring.

To test whether these faster migrating H3 bands were simply artifacts due to suboptimal processing, I first assessed their resilience to protease inhibitors: I made sure to use inhibitors of against all protease classes, increased the concentrations I added to my buffers, tried different “complete” inhibitor formulas from different vendors, and added inhibitors to all my lysis buffers (including the final SDS sample buffer). When these treatments all failed to eliminate the faster-migrating H3 band, I tried the inverse approach: I added recombinant HIS-tagged H3 (rH3-HIS) to 1% SDS lysis buffer and then added this mix to my cells to prepare whole cell extracts (WCEs). When I used these “spike-in” samples to generate immunoblots and then probed them with an anti-H3K27me2 antibody, I still detected the faster migrating H3 band at the same time points (days two and three post-differentiation); however, when I used an anti-HIS antibody to detect the added rH3-HIS, it did not show evidence of clipping (18). Importantly, this rH3-HIS protein later proved to be a robust *in vitro* substrate for H3 clipping activity when I mixed it with cell extracts that preserved it (*i.e.*, no SDS or inhibitors). Moreover, it yielded very similar cleavage products as those detected in endogenous histone preps (18). These results suggest that the enzyme(s) in differentiating ESCs that clips H3 does not require it to be incorporated into the chromatin to perform its activity; they also support the spike-in assay as a reasonable way to surveil for extraction-induced H3 clipping.

Feeling reassured that the H3 clipping I observed was not simply the result of poor sample preparation, I then set out to identify the protease responsible for this activity. As with the isolation of any enzyme from a complex protein extract, this was a daunting task. However, I had the great fortune of being guided by both Dave and our colleague Robert (Bob) Roeder, a world-renowned biochemist and giant in the field of transcription. Bob was particularly helpful, as he vividly remembered the many hours he spent in the cold room fractionating nuclear extracts earlier in his career and also seemed to thoroughly enjoy discussing the specific details of buffer components and column resins. Bob’s insight and advice were particularly critical in helping me think about how to liberate the H3 clipping activity from the chromatin of differentiating cells. As I worked to develop an assay with recombinant H3 and differentiating ESC extracts, I noticed that it was difficult to solubilize clipping activity using standard salt extraction methods. Bob pointed out that this is also true for active RNA polymerase II, which is tightly associated with the chromatin template (43, 44). After testing a few different strategies, I found success in applying solubilized chromatin to a column of hydroxyapatite (45). This allowed me to simultaneously extract and fractionate chromatin-associated proteins from the DNA, which stayed bound to the hydroxyapatite (45). I then tested each fraction in my H3 clippase assay to identify those that contained H3 clipping activity and shipped the active fractions to our mass spectrometry collaborators in the Hunt Laboratory (University of Virginia).

After receiving the results of the mass spec analysis, I was initially disappointed. Although Tara (Muratore-Schroeder, the Hunt Laboratory graduate student with whom I was collaborating) had identified many proteins that were specific to my active fractions, none of them were known proteases. Thankfully, however, Tara had documented the specific details of her analysis in her report, including the fact that she had mapped the peptides from my samples to a human proteome database rather than a mouse one. Weary of cloning and testing dozens of poorly annotated genes in the hope that one might be an unknown protease, I called Tara to confirm this information was correct and ask why she had not used a mouse proteome, as my extracts were from mouse ESCs. She said they often used the human database by default, as the proteomes of the two mammals are so similar, but would re-run the analysis using a mouse-specific database to be thorough. Excitingly, this new analysis identified Cathepsin L (CTSL), a well-known cysteine protease, as one of the proteins in my active fractions. I was both relieved and encouraged; not only was CTSL a known protease but also one that was reportedly (46) able to cleave H3 at the exact site I had mapped. Most importantly, although this enzyme is best known as a lysosomal protease, it had recently been shown to localize to the nuclei of mammalian cells during S phase (47, 48, 49). After years of tedious work, we finally had a promising lead in our search for the H3 clippase.

We then performed several follow up experiments to validate our findings. First, we used recombinant CTSL in our *in vitro* assay to show that it created a highly similar pattern of cleavage products as the endogenous ones we extracted from differentiating ESCs (which we confirmed by mass spec). Next, we used RNA interference (RNAi) to knockdown *Cstl* expression and show that it led to a reduction in clipped H3. Finally, we used a CTSL-specific inhibitor to show that H3 clipping is reduced in cells that were treated with this molecule. Notably, these experiments also revealed that the precise H3 clipping pattern created by CTSL is strongly influenced by the context of the interaction, such as the pH and the posttranslational modification (PTM) status of the H3 tail (18). These details and others hinted at possible regulatory mechanisms of CTSL activity.

Another finding that suggested Cathepsin L activity may be regulated *in vivo* was our mass spectrometry analysis of clipped histones extracted from differentiating stem cells; most strikingly, we only detected clipped forms of the replication-dependent histone protein H3.2, not its replication-independent variant H3.3 (18). This was surprising, as the only sequence difference between H3.2 and H3.3 in the N-terminal tail is at amino acid 31 (A31 in H3.2, S31 in H3.3). Moreover, our lab had recently shown that the percentage of H3.3 protein is robust in this line of ESCs and in fact increases during retinoic acid (RA) differentiation (50). Nevertheless, the clipped H3 protein detected in our mass spec analysis all contained an alanine at position 31. At the same time, follow-up experiments showed that Cathepsin L can clip both H3 variants *in vitro* *i.e.*, rH3.2-HIS and rH3.3-HIS, without obvious preference (E.M.D. personal communication). These data suggest there are likely additional factors regulating the H3 clipping activity of Cathepsin L *in vivo*, restricting it to certain H3 variants and/or loci. One possible mechanism for this regulation is through histone PTMs. For example, *in vitro* assays with Cathepsin L and chemically modified histones showed that lysine acetylation has a strong inhibitory effect on H3 clipping. Moreover, mass spectrometry of endogenous clipped H3 detected many peptides with H3K27 methylation but none with acetylation at this site (18).

As described earlier, my colleagues and I strongly considered the possibility that H3 clipping activity might be an artifact of processing. Although our testing of this hypothesis indicated it was not, it is important to acknowledge that the authors of at least one other study using multiple human cell lines have come to the opposite conclusion (51). In their experiments, they showed that increased cell density (or confluency) correlated with increased CTSL expression and its cleavage products, including clipped H3 (51). Further, they found that the addition of CTSL inhibitors to their lysis buffers dramatically reduced the detection of these cleavage products (51). These findings may indeed point to a common cause of clipping artifacts in nuclear extracts. However, they do not necessarily explain H3 clipping in all cell types and contexts. In our experiments, we detected similar levels of clipped H3 in both whole-cell extracts (made by lysing cells directly in sample buffer containing both 1% SDS and protease inhibitors) and in acid extracted histones. Moreover, I detected clipped H3 at a time point when my differentiating stem cells were in fact *less* confluent than undifferentiated cells (in which I did not detect clipping). As many cells die during RA differentiation, this prompted us to ask if H3 clipping was mainly occurring in a subset of cells undergoing apoptosis. However, I did not detect established markers of apoptosis or DNA damage *i.e.*, cleaved caspase-3 or γ-H2A.X, in my samples (E.M.D. thesis (52)). Together, these data reflect how difficult it is to distinguish between *in vivo* histone clipping and that which was unintentionally permitted during processing, especially when both may be present in the same sample.

One hypothesis that may explain the appearance of low, but reproducible, levels of clipped H3 in differentiating ESCs is that nuclear CSTL activity is regulated by the cell cycle. Although I did not test this hypothesis in my own experiments, other lines of evidence support it: first, Dave’s experiments in *Tetrahymena* show that H3 clipping is only induced in growth conditions. Second, a recent study used optimized CSTL activity-based probes to show that CSTL traffics into the nucleus of mammalian cells immediately before they undergo mitosis (1). In a population of asynchronously dividing cells, this suggests that only a fraction of cells would be permissive to H3 clipping. Notably, I did not detect clipped H3 in undifferentiated ESCs, even though they are rapidly dividing. Yet CSTL expression was undetectable in these cells and increased significantly after the induction of differentiation with RA (18). Overall, these data suggest that *in vivo* H3 clipping is highly context-dependent and potentially limited by the chromatin, differentiation, and/or cell cycle state.

## H3 clipping during tissue-specific differentiation

Adding support to the hypothesis that H3 clipping is developmentally regulated during cell differentiation, and potentially playing a functional role in these processes, this activity has also been reported to occur in the later stages of osteoclast differentiation (53) and *in vivo* intestinal cell differentiation (12). In the former, histone clipping appears restricted to H3 protein (*versus* H2A, H2B, or H4) and is generated by the matrix metalloproteinase MMP-9 (53). Cleavage occurs between H3K18 and H3Q19, a site near but not identical to the site of CSTL cleavage in differentiating ESCs (18). In the intestine, H3 clipping is robustly detected in the villi and but not observed in the crypt (12). Clipping in the intestine is not specific to histone H3, as H2B and H4 also show evidence of proteolysis (but not H2A). H3 is also clipped at more sites than the other histones, with at least three truncated H3 bands detected in cells from the intestinal villi. This is likely because multiple proteases are mediating this H3 clipping, including Trypsin enzymes and CSTL (12).

Interestingly, histone PTMs appear to play a role in modulating H3 clipping activity in both osteoclastogenesis and intestinal differentiation. H3 clipping by MMP-9 was found to be enhanced by H3K18 acetylation and reduced by H3K23 acetylation *in vitro* (53), effects I also observed with rCSTL (18). The three H3 cleavage products detected in the intestinal villi were also shown to have distinct PTM patterns. For example, the product formed by cleaving between H3R8 and H3K9 is enriched for H3K14ac, H3K18ac, and H3K27ac, but not H3K27me3. However, the product created by cleaving between H3R26 and H3K27 is highly modified with H3K27me3 but not lysine acetylation. These different patterns may reflect several different regulatory mechanisms, especially as multiple proteases are implicated in clipping H3 in these villus cells. Nevertheless, they still support the broad hypothesis that histone PTMs influence protease activity and may reflect the existence of regulatory mechanisms that restrict histone cleavage to particular cell types and states.

## A new era of histone clipping studies: aging and cancer

After a long lull in histone clipping research, there has been a significant upsurge of papers in the last decade that report H3 clipping in multiple cell types and physiological contexts (2, 3, 5, 8, 9, 10, 11, 12, 13, 54, 55, 56, 57, 58, 59). The two dominant themes that have emerged are its roles in cellular senescence and oncogenesis. For example, a link between CSTL clipping of H3 and the transcriptional changes needed to drive cellular senescence has been observed in both an oncogene-induced model of fibroblast senescence (2, 59) and hematopoietic stem cells after deletion of the *Bromodomain containing 4* (*Brd4*) gene (5). These data suggest that H3 clipping is a protective response to oncogenic events and that this response may be inhibited by overexpression of BRD4, a common feature of various cancers (60, 61, 62, 63, 64, 65, 66). At the same time, other studies have shown that histone clipping is pro-tumorigenic; H3 clipping by Matrix Metalloproteinase 9 (MMP-9) is a consistent feature in both multiple colon cancer cell lines and melanoma cells, likely through the activation of growth-associated genes (3, 9). Moreover, targeting MMP-9 to gene promoters in MMP-9 depleted colon cancer cells was shown to restore growth-related transcription, cell proliferation, and colony formation (3). These studies suggest that H3 clipping can have opposing roles in the progression of cancer depending on the cell type and mutational signature.

Further investigation has uncovered at least one context that may regulate histone clipping during oncogene-induced senescence (OIS) (2). Fibroblasts incubated in hypoxic conditions (0.5% oxygen) exhibited less histone clipping and fewer markers of senescence after the induction of oncogene expression than cells undergoing the same transformation but cultured in normoxic conditions (21% oxygen) (2). These researchers also observed that fibroblasts cultured in hypoxic conditions had increased levels of both H318 and H3K23 trimethylation, which shielded its N-terminal tail from CSTL clipping (2). These experiments reinforce the model in which the chromatin state regulates clippase activity, even though the specific effects of particular PTMs depend on the exact protease and cellular context. Moreover, they also point to the importance of testing how different physiological and/or pathological conditions affect the chromatin state.

## Future directions

Clipped, or proteolyzed, histones have been detected in the nuclear extracts of many different tissues from many different organisms and in many different contexts, yet their causal roles remain enigmatic. One reason for this inscrutability is that the proteases responsible for their clipping have multiple protein substrates, making it hard to determine if histone clipping itself is biologically important. In addition, most organisms have many copies of histone genes in their genomes, making it extremely difficult to test substrate-specific function by mutating a particular cleavage site in the histone tail. Data from such experiments in budding yeast, a model in which it is possible to mutate the cleavage site in all H3 proteins, shows that H3 clipping does indeed induce gene activation (67). Although not conclusive, this adds support to the growing collection of correlative data that suggests histone clipping affects transcription in mammalian cells. As interest in the role of histone clipping continues to grow, it will be important to find solutions to this technical hurdle and perform more experiments that examine the roles of substrate and site-specific cleavage.

In the meantime, the data described here represents a growing yet valuable resource that raises many interesting questions. For example, what are the regulatory mechanisms that trigger and/or permit histone clipping during the differentiation of various cell lineages? Are these mechanisms linked to those that regulate the cell cycle? How do proteases access the nucleus/chromatin and how is this regulated? Particularly given the increasing evidence suggesting a link between histone clipping and cancer, it is important that we understand the cell biology and molecular mechanisms of this activity. In addition, these data inspire me to wonder if we can use histone proteases in assays of chromatin state, particularly given that different proteases generate distinct cleavage products and their precise activities are affected by chromatin PTMs.

Another avenue for major discovery in this field will be the examination of histone clipping activity in more cell types and organisms. For example, a recent study in the malaria parasite *Plasmodium falciparum* found that its histone H3 (Pf-H3) is clipped in a developmental stage-specific manner and at a cleavage site (between A21 and S22) that is reminiscent of the clipping seen in mouse ESCs (54). The authors also provide evidence that a Cathepsin-like protease may be responsible for this clipping. Additionally, this study included an experiment that tested whether the clipped Pf-H3 protein can be incorporated into the chromatin in its truncated form *i.e.*, post-clipping (54). As also shown in senescent mammalian cells (5, 59), their data revealed that exogenously expressed clipped Pf-H3 was successfully incorporated into the chromatin and, moreover, at specific gene loci. Although the authors did not perform gene expression studies to assess the effects of clipped Pf-H3 incorporation on transcription, they did find that many of these genes share a common function *i.e.*, the proteins they encode regulate DNA replication. Undoubtedly, additional studies are needed to address whether this locus-specific incorporation of clipped Pf-H3 reflects an endogenous mechanism and if it has a functional role in *P*. *falciparum* biology. Nevertheless, this work adds to an expanding collection of data (Fig. 1) that suggests histone clipping may be an evolutionarily conserved mechanism to regulate chromatin function.

Until recently, relatively few researchers studied histone clipping, making it hard for the field to gather the momentum needed to address these questions. Although the loss of David Allis has left the field without one of its strongest advocates, I hope I have imbued this review with his deep interest and cautious excitement about the potential significance of histone clipping. The discovery of regulated histone clipping in *Tetrahymena* micronuclei is only a small part of Dave’s incredible legacy to chromatin biology, but it is one that he loved revisiting. He would undoubtedly invite any curious newcomers to join this field and encourage them to help us tackle these intriguing, yet unanswered, questions.

E.M.Duncan is the sole author of this review and is responsible for the conceptualization, writing, revision, and figure generation.

## Conflict of interests

The authors declare that they have no conflicts of interest with the contents of this article.
